# Differences in Immune-Related Genes Underlie Temporal and Regional Pathological Progression in 3xTg-AD Mice

**DOI:** 10.3390/cells11010137

**Published:** 2022-01-01

**Authors:** Adelaide Fernandes, Cláudia Caldeira, Carolina Cunha, Elisabete Ferreiro, Ana Rita Vaz, Dora Brites

**Affiliations:** 1 Central Nervous System, Blood and Peripheral Inflammation, Research Institute for Medicines (iMed.ULisboa), Faculdade de Farmácia, Universidade de Lisboa, 1649-003 Lisboa, Portugal; 2 Department of Pharmaceutical Sciences and Medicines, Faculdade de Farmácia, Universidade de Lisboa, 1649-003 Lisboa, Portugal; armvaz@ff.ulisboa.pt; 3 Neuroinflammation, Signaling and Neuroregeneration, Research Institute for Medicines (iMed.ULisboa), Faculdade de Farmácia, Universidade de Lisboa, 1649-003 Lisboa, Portugal; claudiacaldeira@mail.telepac.pt (C.C.); jcarolina.cunha@gmail.com (C.C.); 4 Bruno Silva-Santos Lab, Instituto de Medicina Molecular, Faculdade de Medicina, Universidade de Lisboa, 1649-003 Lisboa, Portugal; 5 MitoXT-Mitochondrial Toxicologu and Experimental Therapeutics Laboratory, CNC-Center for Neuroscience and Cell Biology, Universidade de Coimbra, 3004-516 Coimbra, Portugal; ebf@cnc.uc.pt; 6 III-Institute for Interdisciplinary Research (IIIUC), Universidade de Coimbra, 3004-516 Coimbra, Portugal

**Keywords:** Alzheimer’s disease, APP processing, dysregulated gene-associated biomarkers, inflammatory-associated miRNAs, microglia reactivity, miR-155 targets, 3xTg-AD mouse model

## Abstract

The prevalence of Alzheimer’s disease (AD), the most common cause of age-associated dementia, is estimated to increase over the next decades. Evidence suggests neuro-immune signaling deregulation and risk genes beyond the amyloid-β (Aβ) deposition in AD pathology. We examined the temporal profile of inflammatory mediators and microglia deactivation/activation in the brain cortex and hippocampus of 3xTg-AD mice at 3- and 9-month-old. We found upregulated APP processing, decreased expression of *CD11b*, *CX3CR1*, *MFG-E8*, *TNF-α, IL-1β, MHC-II* and *C/EBP-α* and increased miR-146a in both brain regions in 3-month-old 3xTG-AD mice, suggestive of a restrictive regulation. Enhanced *TNF-α*, *IL-1β*, *IL-6*, *iNOS*, *SOCS1* and *Arginase 1* were only present in the hippocampus of 9-month-old animals, though elevation of *HMGB1* and reduction of miR-146a and miR-124 were common features in the hippocampus and cortex regions. miR-155 increased early in the cortex and later in both regions, supporting its potential as a biomarker. Candidate downregulated target genes by cortical miR-155 included *Foxo3*, *Runx2* and *CEBPβ* at 3 months and *Foxo3*, *Runx2* and *Socs1* at 9 months, which are implicated in cell survival, but also in Aβ pathology and microglia/astrocyte dysfunction. Data provide new insights across AD state trajectory, with divergent microglia phenotypes and inflammatory-associated features, and identify critical targets for drug discovery and combinatorial therapies.

## 1. Introduction

Alzheimer’s disease (AD) is, nowadays, the most common cause of dementia in elderly people, and, due to their complexity, no drugs have proven efficacy. However, the testing of some new treatments, now in the late stage of clinical development, may originate novel therapeutic approaches. A better understanding of the causes behind AD and an early diagnosis can open the door for future care and therapeutics. To achieve this goal, more than 150 AD animal models were developed according to AlzForum. Several mouse models have been generated to recapitulate behavior and neuropathology like that observed in AD patients. In this context, mouse models with mutations in more than one gene are considered to better mimic AD pathogenesis than those only reflecting single aspects [1]. 

To date, commonly used animal models are transgenic mice that overexpress human genes associated with familial AD, leading to the formation of senile plaques of amyloid-β (Aβ) peptide (e.g., overexpression of human amyloid precursor protein (APP)) alone or with presenilin-1 (PSEN1) and neurofibrillary tangles of hyperphosphorylated tau protein (e.g., overexpression of human microtubule associated protein tau (MAPT)), two hallmarks of AD [2]. Recently, the validity of such models has been questioned by the high failure rate of clinical trials of AD therapeutics (>99%) that were successful in preclinical trials using these models [3]. Thus, to enhance translational predictability, new models should be developed to incorporate genetics with multiple factors to further advance the molecular and cellular markers that trigger familial and sporadic AD-related cognitive decline [1,4]. Nevertheless, the data generated by experimental AD animal models can bring new highlights about specific aspects of AD pathogenesis if researchers consider the limitations of each model. 

Although the introduction of wild-type forms of APP and PSEN1, as found in normal and non-familial AD cases, does not elicit AD pathology in mice, the transgenic mice overexpressing pathological human mutant proteins easily show Aβ aggregation, cognitive deficits, and some abnormal tau phosphorylation [5,6,7]. The mouse model APP/PS1ΔE9 that expresses *APP* with the Swedish mutation and mutant human *PSEN1* with a deletion of exon 9 shows formation of amyloid plaques prior to typical cognitive impairments [8,9], is used to examine pathophysiological events associated to preclinical AD [10]. Another useful model of intraneuronal Aβ-induced neurodegeneration is the 5xFAD [11]. This mouse model expresses five familiar AD mutations, two for *PSEN1* and three for *APP*, that induce Aβ overproduction to accelerate plaque development [12]. Consequently, 5xFAD mice develop intraneuronal Aβ at 1.5 months of age, amyloid plaques at 2 months and significant neuron loss at 9 months [12]. However, these models do not exhibit the development of the neurofibrillary tangles characteristic of the human disease [13]. On the other hand, the triple-transgenic mice model (3xTg-AD) progressively develops both Aβ plaques and neurofibrillary tangles with a temporal and spatial distribution that recapitulates the disease in humans [14,15]. These animals contain three major mutations associated with AD (*APPSwe*, *PSEN1M146V* and *tauP301L*), leading to intraneuronal Aβ accumulation first in the cortical region that later spreads into hippocampus, followed by the emergence of neurofibrillary tangles in the hippocampus that disseminate into cortical regions [14,15]. In previous studies conducted in the Center for Neuroscience and Cell Biology (CNC) at Coimbra, Portugal, the development of amyloid and tau pathologies in the 3xTg-AD mouse males was previously identified [16,17]. No differences in the number and phenotype of microglia and astrocytes, using the ionized calcium-binding adapter molecule 1 (IBA-1) and the glial fibrillary acidic protein (GFAP) assessment by immunohistochemistry, respectively, were identified in these animals at 3-month-old [16]. However, extensive microglia and astrocyte proliferation in the hippocampus and cortex of the 3xTg-AD mice were observed at 12 months. It should be noted that Aβ intraneuronal immunostaining was already found to be significantly enhanced in the hippocampus and prefrontal cortex in the mice at 3-month-old.

In these animals, behavioral alterations were identified as early as 2.5 months [18,19] and cognitive decline was detected at 4 months, when intraneuronal Aβ accumulated in the cortex, hippocampus and amygdala [20], which is consistent with the amyloid cascade hypothesis. Interestingly, the 3xTg-AD animals also showed extensive astrogliosis and microgliosis with upregulation of inflammatory molecules after 6 months [21,22], which occurs prior to neurofibrillary tangle detection.

An elevated number of activated astrocytes and microglia are usually found close to neurons and Aβ plaques [23]. Microglia, the resident immunocompetent and phagocytic cells in the central nervous system (CNS) [24], can be either neuroprotective or neurotoxic, thus promoting a decrease or worsening of disease progression, respectively [25]. In fact, while microglia phagocytic capacity contributes to CNS neuroprotection from excessive Aβ, microglial activation by Aβ soluble oligomers promotes excitotoxicity and neurodegeneration by the release of several inflammatory cytokines, such as tumor necrosis factor (TNF)-α and interleukin (IL)-1β, contributing to the onset and progression of AD [26,27]. Most curiously, single cell RNAseq studies using the 5xFAD mice and AD patient samples identified a novel microglia type associated with neurodegenerative diseases (DAM), the activation of which involved an initial downregulation of microglia checkpoints [28]. In addition, a dystrophic microglia phenotype was also identified in AD patient autopsied samples [29]. 

We have previously addressed changes in microglia reactivity with age and Aβ challenge using an in vitro aging model developed in our laboratory [30]. We showed that several neuroprotective functions, including phagocytosis, migration abilities and autophagy, are impaired by in vitro aging [31], while the Aβ-induced inflammatory response is also reduced in aged cells with the compromised expression of miRNAs associated with inflammatory responses (inflamma-miRNAs) [30]. miRNAs, a class of small, non-coding RNAS, are important players in controlling inflammation and have a key regulatory role in microglia polarization [32]. Most attractively, we demonstrated that exposure of young/reactive microglia to Aβ promotes cellular senescence [30], in accordance with the link between AD and immunosenescence [33]. Changes in several microglia inflammatory mediators, including inducible nitric oxide synthase (*iNOS*), *TNF*-α, *IL-1β*, high mobility group box 1 (*HMGB1*), suppressor of cytokine signaling 1 (*SOCS1*) and *Arginase 1* gene expression levels, were found to be deregulated in our previous in vitro AD studies and aged-like microglia model [30,34].

In the present study, we decided to explore microglia reactivity and inflammatory mediators in the cortex and hippocampus of the 3xTg-AD at 3-, 6- and 9-month-old mice, which are temporal stages also used in other studies [35]. For that, we evaluated the mRNA expression of specific markers of microglia reactivity, namely functional genes, inflammatory cytokines, phenotype-related molecules, and inflamma-miRNAs. We report a reduction of *CD11b*, *C-X3-C motif chemokine receptor 1* (*CX3CR1*) and *milk fat globule-EGF factor 8* (*MFG-E8*) gene expression levels in the cortex and hippocampus of 3-month-old 3xTg-AD animals, as well as a decrease of inflammatory cytokines (*TNF-α* and *IL-1β* in both regions and *IL-6, IL-18* and HMGB1 only in the brain cortex). Downregulation of the *major histocompatibility complex class II* (*MHC-II*) and *CCAAT enhancer binding protein* (*C/EBP*)-α pro-inflammatory markers was additionally observed in both brain regions, together with the anti-inflammatory *SOCS1*, *transforming growth factor* (*TGF*)-β and *Arginase 1* genes, as well as with deregulated anti-inflammatory cytokines (*IL-10* and *IL-4*) in the brain cortex. Increased levels of *HMGB1* (both regions), of *CX3CR1* (brain cortex) and of *iNOS*, *TNF-α, IL-1β, IL-6, SOCS1* and *Arginase 1* (hippocampus) were present in 9-month-old 3xTg-AD animals. We also provide evidence that miR-155, miR-124 and miR-146a were upregulated, mainly in the cortex of 3-month-old 3xTg-AD mice. Collectively, our data show an early dysfunctional/constrained microglia immune response that may represent a preventive intervention to hold the onset of the disease in the younger transgenic mice. However, with the appearance of homeostatic imbalance at 9 months, microglia in the 3xTg-AD mice develop an inflammatory response and lose their immune neuroprotective properties. In summary, according to our results, the 3xTg-AD mice at 3- month-old are a good model to explore the pathomechanisms that precede AD onset and to test novel therapeutic strategies towards balance and homeostasis. 

## 2. Materials and Methods

### 2.1. Animals

Animal care followed the recommendations of the European Convention for the Protection of Vertebrate Animals Used for Experimental and Other Scientific Purposes (Council Directive 86/609/EEC) and National Law 1005/92 (rules for the protection of experimental animals). All animal procedures were approved by the Institutional Animal Care and Use Committee. Every effort was made to minimize suffering and the number of animals used in this study.

**Figure 1:**
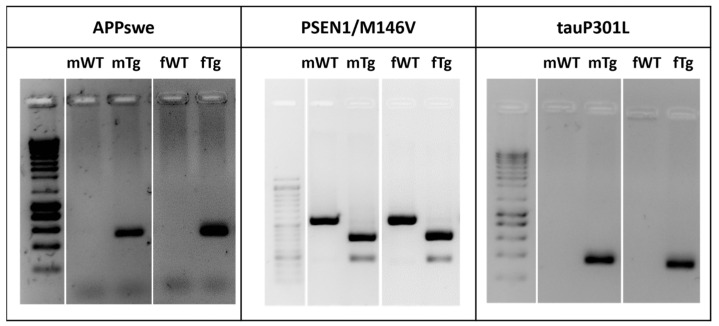
3xTg-AD mice harbor *APPswe*, *PSEN1M146V* and *tauP301L* transgenes. Genotypic analysis for identification of 3xTg-AD mice. Representative gels comparing the human *APPswe*, *PS1/M146V* and *tauP301L* transgenes from tail DNA of breeding pairs (m—male and f—female) of WT and 3xTg-AD mice.

Breeding pairs of homozygous triple-transgenic AD (3xTg-AD) mice harboring *PSEN1M146V*, *APPswe* and *tauP301L* transgenes and age-matched non-transgenic mice with the same genetic background (C57BL/6 × 129 s), denominated as wild-type (WT) animals and used as controls, were kindly provided by Dr. Frank LaFerla from the University of California, Irvine (Irvine, CA, USA). Animals were genetically engineered as previously described [14,15]. Mice were bred and maintained at the CNC Faculty of Medicine animal house (license nº 520.000.000.2006, from the Portuguese animal welfare authorities), University of Coimbra, Portugal. The animals were maintained under controlled light and environmental conditions (12 h dark/light cycle, 23 ± 1 °C, 55 ± 5% relative humidity), having access to food and water, and breeding pairs were continuously genotyped for the detection of *PSEN1M146V*, *APPswe* and *tauP301L* transgenes, as shown in Figure 1. These 3xTg-AD mice did not show changes in IBA-1 or GFAP immunostaining at 3 months but revealed intraneuronal Aβ increase in the hippocampus and prefrontal cortex, as well as extensive microglia and astrocyte proliferation, at 12-month-old [16]. Based on such data and given our interest to identify early biomarkers of neuro-immune deregulation and homeostatic imbalance in the 3xTg-AD mouse model, we decided to assess potential glia-associated pathological drivers in male animals at 3-, 6- and 9-month-old. The animals were euthanized, and the brains were removed following transcardial perfusion with 20 mL of an ice-cold 0.9% NaCl solution. The brain hemispheres were separated and used for protein and mRNA extraction. For this purpose, each hemisphere was placed on an acrylic matrix and a 4 mm coronal section was cut with a stainless-steel razor. The hippocampal and cortical regions from this section were dissected and kept at −80 °C until protein and mRNA extraction.

### 2.2. Evaluation of APP/Aβ Peptide and HMGB1 Expression

Detection of APP/Aβ protein and HMGB1 expression levels was processed by Western blot as usual in our laboratory [34]. Total protein extracts were obtained from brain extracts collected from the cortex and hippocampus of 3xTg-AD mice and their WT littermates. Briefly, tissues samples were lysed using TRIzol^®^ (Life Technologies, Carlsbad, CA, USA), according to manufacturer’s instructions. Protein extracts were obtained as previously described [36] with minor alterations and stored at −80 °C. Protein concentrations were determined using BioRad protein assay (Bio-Rad, Hercules, CA, USA). Cell extracts containing equal amounts of protein (100–150 µg) were separated on a 10 to 15% sodium dodecyl sulfate-polyacrylamide gel electrophoresis (SDS-PAGE) system and transferred to a nitrocellulose membrane. The membranes were blocked with 5% non-fat milk and incubated with the primary antibody mouse anti-Aβ (clone W0-2) antibody (1:500, SIGMA-Aldrich, St. Louis, MO, USA) that detects both APP and Aβ species, mouse HMGB1 (1:200, BioLegend, San Diego, CA, USA) and mouse β-actin (1:5000, SIGMA-Aldrich) overnight at 4 °C and then with horseradish peroxidase-labelled secondary antibodies for 1 h at room temperature. After extensive washes with saline buffer, immunoreactive bands were detected by LumiGLO^®^ (Cell Signaling, Beverly, MA, USA) and visualized by chemiluminescence with ChemiDoc (Bio-Rad). Expression of HMGB1 expression was quantified by computerized image analysis using the Quantity One 1-D Analysis Software (Bio-Rad). Results were normalized to β-actin expression and expressed as fold vs. WT brain cortex from 3-month-old mice.

### 2.3. Gene and miRNA Expression Profiling

Determination of mRNA expression was performed by RealTime quantitative PCR (RT-qPCR) as usual in our laboratory [34]. Total RNA was extracted from hippocampal and cortical tissues of 3xTg-AD and WT animals using TRIzol^®^ (LifeTechnologies), according to manufacturer’s instructions. Total RNA was quantified using Nanodrop ND-100 Spectrophotometer (NanoDrop Technologies, Wilmington, DE, USA). Aliquots of 1 µg of total RNA were treated with DNase I and then reverse transcribed into cDNA using oligo-dT primers and SuperScript II Reverse Transcriptase under the recommended conditions. RT-qPCR was performed using β-actin as an endogenous control to normalize the expression levels of transcription factors. The sequences used as primers are represented in Table 1. RT-qPCR was accomplished on a RT-PCR detection system (Applied Biosystems 7300 Fast Real-Time PCR System, Applied Biosystems, Madrid, Spain) using the SensiFAST SYBR^®^ High-ROX kit (Bioline, Toronto, ON, Canada). The PCR was carried out in 96-well plates with each sample performed in triplicate, and no-template control was included for each amplification product. The RT-qPCR protocol was carried out under optimized conditions: 50 °C for 2 min followed by 95 °C for 2 min and, finally, 40 cycles at 95 °C for 0.05 min and 62 °C for 0.30 min. To verify the specificity of the amplification, a melt-curve analysis was performed immediately after the amplification protocol. Non-specific products of PCR were not found in any case. The results were normalized to β-actin in the same sample and relative mRNA concentrations calculated by the formula 2^−ΔΔCT^, considering 100% efficiency for each gene. ΔC_T_ was the value obtained for each sample by assessing the difference between the mean C_T_ value of each gene and the mean C_T_ value of β-actin. ΔΔC_T_ of one sample was the difference between its ΔC_T_ value and the ΔC_T_ of the sample chosen as reference, in our case the cortex of 3-month-old WT mice. 

For miRNA analysis, cDNA conversion was performed with the universal cDNA Synthesis Kit (Qiagen, Hilden, Germany), as usual in our lab [34], using 5 ng total RNA according to the following protocol: 60 min at 42 °C followed by heat inactivation of the reverse transcriptase for 5 min at 95 °C. RT-qPCR was performed as previously indicated. For miRNA quantification, the miRCURY LNA™ Universal RT microRNA PCR system (Qiagen) was used in combination with pre-designed primers (Qiagen) for miR-155, miR-124, miR-146a and SNORD110 (reference gene) (Table 2). The reaction conditions consisted of polymerase activation/denaturation and well-factor determination at 95 °C for 10 min, followed by 50 amplification cycles at 95 °C for 10 s and 60 °C for 1 min (ramp rate 1.6°/s). The miRNA fold change with respect to the cortex of 3-month-old WT mouse samples was determined by the Pfaffl method, considering different amplification efficiencies of miRNAs in all experiments. The amplification efficiency for each target or reference RNA was determined according to the formula: *E* = 10^(−1/*S*)^ – 1, where *S* is the slope of the obtained standard curve.

### 2.4. Assessment of miR-155 Target Expression

To assess the expression of genes regulated by miR-155, total RNA was extracted from cortical tissue of 3xTg-AD and WT animals and quantified as described above. A 500-ng amount of total RNA of each animal was pooled and mixed according to age (3 and 9 months) and disease condition (WT vs. 3xTg-AD). Aliquots of 500 ng of total mixed RNA were reverse transcribed into cDNA using the RT^2^ First Strand Kit (Qiagen, Hilden, Germany), according to manufacturer’s instructions. RT-qPCR was performed on a RT-PCR detection system (QuantStudio™ 7 Flex Real-Time PCR System, Applied Biosystems, Madrid, Spain) using the RT^2^ Profiler PCR Array 384-Well (4 × 96) Format (Qiagen) and a RT^2^ SYBR^®^ Green qPCR Mastermix (Qiagen). The PCR was carried out in 384-well plates, and reverse transcription control (RTC) and positive PCR control (PPC) were included to determine the reverse transcription efficiency and PCR reaction, respectively. The results were normalized to a standard set of reference genes. The threshold cycle (C_T_) values were analyzed with the RT^2^ Profiler PCR Array Data Analysis Webportal and geometrically averaged and used for ΔΔC_T_ calculations. Fold change was calculated by using ΔΔC_T_ method [37], corresponding to the ratio of gene expression between the reference or control group (WT) and test group. Fold regulation was used to better read and interpret data. If the fold change values are greater than 1, the fold regulation and fold change values are the same and indicate upregulated or increased gene expression. Fold change values less than 1 imply that fold regulation is the negative inverse of the fold change and indicate downregulated or decreased gene expression. The results were presented in a heat map and further detailed in a scatter plot comparing the normalized expression of each gene between two groups (WT vs. 3xTg-AD). Gene-specific 2^–ΔC_T_^ value in reference group was plotted on one axis against the corresponding value in test group on the other axis on a log base 10 scale to observe gene expression changes. Boundary lines were used to allow better visualization of upregulated and downregulated genes above and below a selected fold change value. The central line indicates unchanged gene expression. The section of the scatter plot above the fold change boundary lines contains genes upregulated in the *y*-axis group as compared to the *x*-axis group, and the section of the scatter plot below the fold change boundary lines contains genes downregulated in the *y*-axis group as compared to the *x*-axis group.

### 2.5. Statistical Analysis

Results of at least four different animals per experimental group are expressed as mean ± SEM. Significant differences between the assessed parameters were determined by the two-tailed Student’s *t*-test performed based on equal and unequal variance, as appropriate, using GraphPad Prism^®^ 8.0 (GraphPad Software Inc., San Diego, CA, USA). *p* value less than 0.05 was considered statistically significant.

## 3. Results

### 3.1. The 3xTg-AD Animals Express Upregulated APP from 3 Months Forward

**Figure 2:**
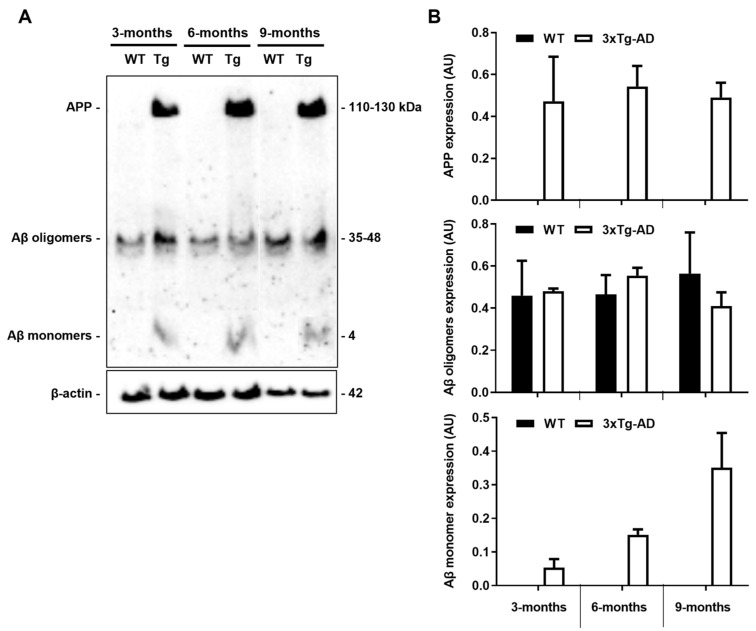
Amyloid precursor protein (APP) and Aβ monomer expression levels are increased in the 3xTg-AD mouse model. Cortical samples from wild-type (WT) and 3xTg-AD animals were collected at 3-, 6- and 9-month-old and analyzed for APP/Aβ expression by Western blot using the Aβ (clone W0-2) antibody. (**A**) Representative image showing APP/Aβ bands. (**B**) Graph bars of the quantitative evaluation of APP/Aβ expression levels (arbitrary units, AU) are mean ± SEM representative of *n* = 3 animals per experimental group.

The 3xTg-AD mouse model, developed in LaFerla’s laboratory, presents cortical intraneuronal Aβ as early as 3 months, followed by cortical extracellular deposits and hippocampal intraneuronal Aβ at 6 months and diffuse plaque formation at 15 months [14]. So, first, we decided to evaluate APP/Aβ protein expression in 3-, 6- and 9-month-old animals before further analysis to be sure that the present cohort of 3xTg-AD mice expressed increased levels of these proteins. Using the Aβ (clone W0-2) antibody that detects both APP and Aβ, we were able to detect the APP species, as well as Aβ oligomers and Aβ monomers, as shown in Figure 2. As expected, we observed a clear expression of APP and Aβ monomers only in the 3xTg-AD mice at 3, 6 and 9 months, but no differences in Aβ oligomers when compared to age-matched WT animals. Although APP expression was not altered by age, Aβ monomers accumulation was age dependent. Since tau phosphorylation was only described in these 3xTg-AD mice at 12 months [14,38], a later time point than those evaluated, we did not assess such protein modifications.

### 3.2. Expression of Microglia Homeostatic/Reactive Genes Is Decreased in the 3xTg-AD Mice at the Early Stage 

Microglia were described to behave differently along AD progression in distinct animal models of the disease associated with Aβ pathology [39,40,41]. Our previous studies showed that microglia aged in vitro become irresponsive even when challenged with Aβ1-42 oligomers and fibrils [30,31]. In this section we decided to assess the temporal expression of microglia common markers in the 3xTg-AD animals. To do that, we evaluated the *CD11b, CX3CR1* and *MFG-E8* gene expression levels in the 3-, 6- and 9-month-old 3xTg-AD animals and age-matched WT mice. While CD11b, a β-integrin marker of microglia, is associated with microglial activation during neuroinflammation [42], CX3CR1 is involved in microglia reactivity [43] with still controversial effects in AD. Relative to MFG-E8, it was reported to be essential for microglia phagocytosis of affected neurons in the presence of Aβ peptide [44].

**Figure 3:**
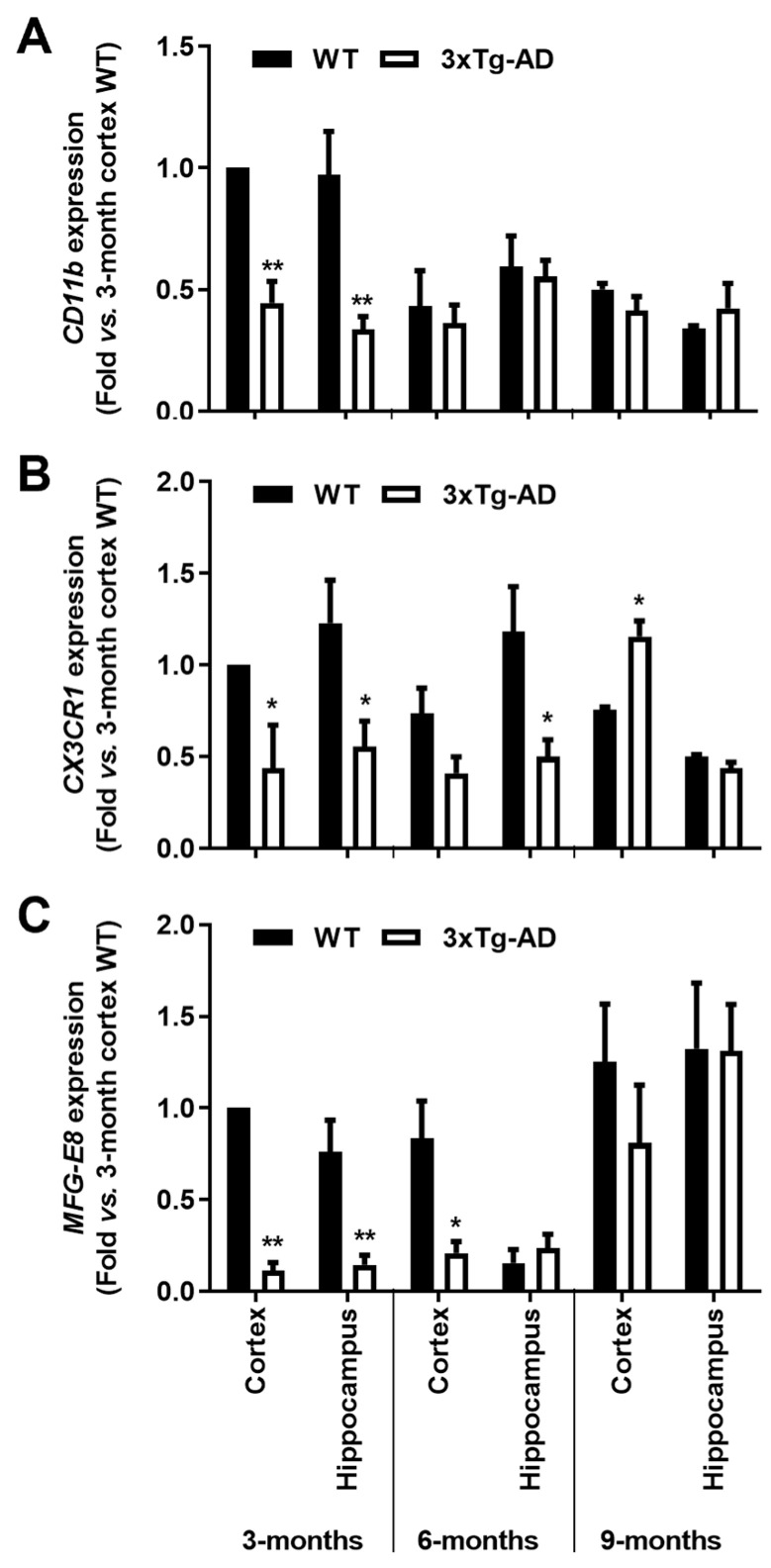
Microglial typical markers are decreased in the brain cortex and hippocampus of the 3-month-old 3xTg-AD mice. Samples from the wild-type (WT) and 3xTg-AD animals were collected at 3-, 6- and 9-month-old and analyzed for (**A**) *CD11b*, (**B**) *CX3CR1* and (**C**) *MFG-E8* mRNAs by RT-qPCR. Results are mean ± SEM, representative of *n* = 4 animals per experimental group, and are expressed as fold change vs. 3-month-old cortex WT mice. * *p* < 0.05 and ** *p* < 0.01 vs. respective WT, two-tailed Student’s *t*-test.

We observed a marked reduction of *CD11b* gene expression levels in the 3-month-old 3xTg-AD animals (~55% in the cortex and ~65% in the hippocampus, *p* < 0.01), together with that of *CX3CR1* (~56% cortex and ~55% hippocampus, *p* < 0.05) and *MFG-E8* (~89% cortex and ~80% hippocampus, *p* < 0.01) that disappeared in older animals (Figure 3). These results are in line with a restrictive microglia phenotype in the early-stage AD mice, suggesting an attempt to hold the inflammatory response to the initial increase of Aβ expression levels in the 3-month-old transgenic mice. 

### 3.3. Expression of Inflammatory Genes in the 3xTg-AD Mice Switch from Downregulated at 3 Months to Upregulated at 9 Months 

Considering that microglia homeostatic/reactive genes were found depressed based on the expression of the *CD11b, CX3CR1* and *MFG-E8* markers, and since inflammatory genes were found downregulated in the *SOD1G93A* mouse model of amyotrophic lateral sclerosis before disease onset [45], we next assessed a set of inflammatory genes associated with neuroinflammation in neurodegenerative diseases. Actually, only a few studies using AD animal models have looked at the early stages of the disease, and, in those cases, pro-inflammatory cytokine expression was found to be most similar to WT animals [38,40] or sometimes even reduced [16,46]. 

Though we assessed samples from 3-, 6- and 9-month-old mice, the 6-month-old period did not reflect gross alterations, probably due to the altered dynamics of intra- and extracellular Aβ deposition at this temporal period [20], and it was excluded from subsequent data presentation.

**Figure 4:**
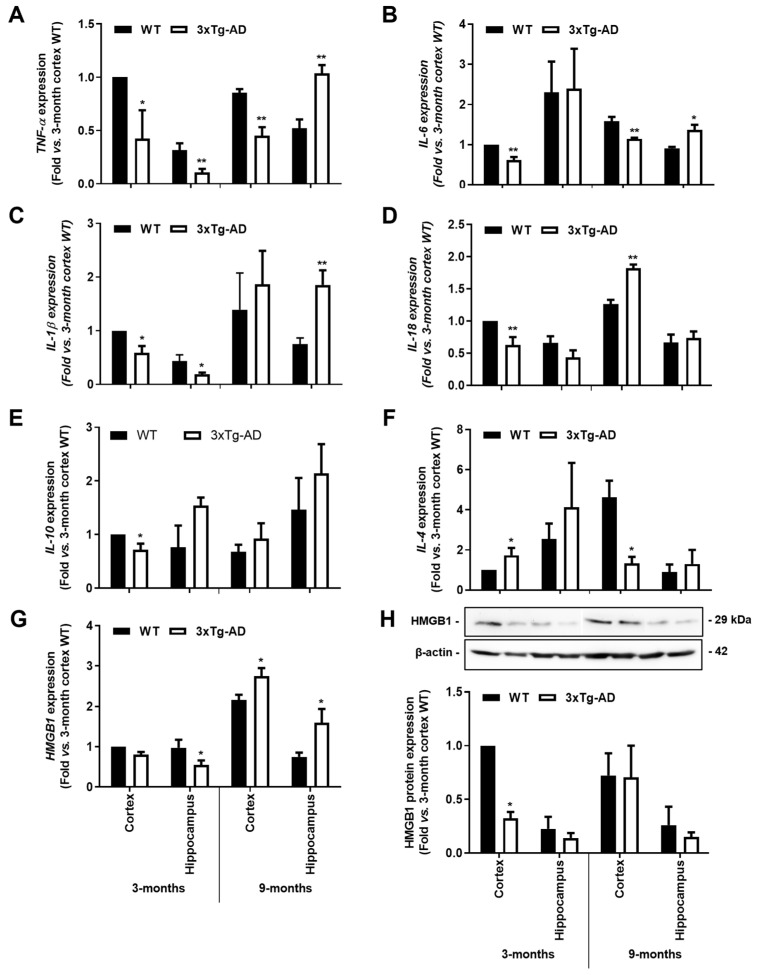
Gene expression levels of pro-inflammatory cytokines are reduced in the 3xTg-AD animal model at the early stage, but some of them increase with disease progression. Brain samples from wild-type (WT) and 3xTg-AD animals were collected at 3 months and analyzed for (**A**) *TNF-α*, (**B**) *IL-6*, (**C**) *IL-1β*, (**D**) *IL-18*, (**E**) *IL-10*, (**F**) *IL-4* and (**G**) *HMGB1* mRNA expression by RT-qPCR, as well as for (**H**) representative Western blot for HMGB1 protein expression levels and respective quantification. Results are mean ± SEM, representative of *n* = 4 animals per experimental group, and are expressed as fold change vs. WT mouse cortex at 3-month-old. * *p* < 0.05 and ** *p* < 0.01 vs. respective WT, two-tailed Student’s *t*-test.

As depicted in Figure 4, all the evaluated pro-inflammatory cytokines showed a generalized reduction of mRNA expression levels in the 3-month-old 3xTg-AD mice when compared to WT animals. In the cortex, *TNF-α*, *IL- 6*, *IL-1β* and *IL-18* expression levels were decreased in the 3-month-old 3xTg-AD animals by more than 40% (*p* < 0.05). From these, *TNF-α* and *IL-6* were still reduced in 9-month-old 3xTg-AD animals, while *IL-18* and *HMGB1* were upregulated. (1.4-fold, *p* < 0.01 and 1.3-fold, *p* < 0.05, respectively). When looking at the hippocampus, *TNF*-α, *IL-1β* and *HMGB1* expression levels were found similarly depressed (*p* < 0.05) in the 3-month-old 3xTg-AD mice, but all were upregulated at 9 months (*TNF-α*, 2-fold, *p* < 0.01; *IL-1β*, 2.4-fold, *p* < 0.01; *HMGB1*, 2.1-fold, *p* < 0.05; and *IL-6*, 1.5-fold; *p* < 0.01). HMGB1 protein expression was found reduced in the 3-month-old 3xTg-AD mice, namely in the cortex (0.3-fold, *p* < 0.05) with no changes in samples from the 9-month-old animals (Figure 4H). Curiously, when evaluating the expression of anti-inflammatory cytokines (Figure 4E,F), while IL-10 was reduced in the cortex of 3-month-old 3xTg-AD mice (0.7-fold, *p* < 0.05), IL-4 expression was enhanced in samples from the same region and age (1.7-fold, *p* < 0.01) but markedly downregulated in the cortex of 9-month-old 3xTg-AD mice (1.3-fold in 3xTg-AD vs. 4.6-fold in WT mice, *p* < 0.05). Data show, for the first time, that the expression levels of inflammatory genes are downregulated early during AD disease in the 3xTg-AD mice, which is in accordance with the low expression of homeostatic and reactive markers demonstrated above. 

### 3.4. Microglia Genes Associated with Inflammatory Programs Differ with Age and Brain Regions in the 3xTg-AD Mice 

It has been suggested that microglia pro-inflammatory polarization is lost in the early stages of AD and precede the occurrence of neuroinflammation. This later effect was demonstrated in AD models, including the APP/PS1, the APP/PS1ΔE9 and the 5xFAD mice [39,40]. Here, we assessed common pro- and anti-inflammatory markers of microglia-polarized subtypes in the cortex and the hippocampus of 3- and 9-month-old 3xTg-AD mice to better define the regional and age-dependent cell phenotypes. For that, we evaluated the mRNA expression of *MHC-II*, *iNOS* and *C/EBP-α*, which are usually considered pro-inflammatory microglial markers and *SOCS1*, *TGF-β* and *Arginase 1*, associated with anti-inflammatory properties. 

**Figure 5:**
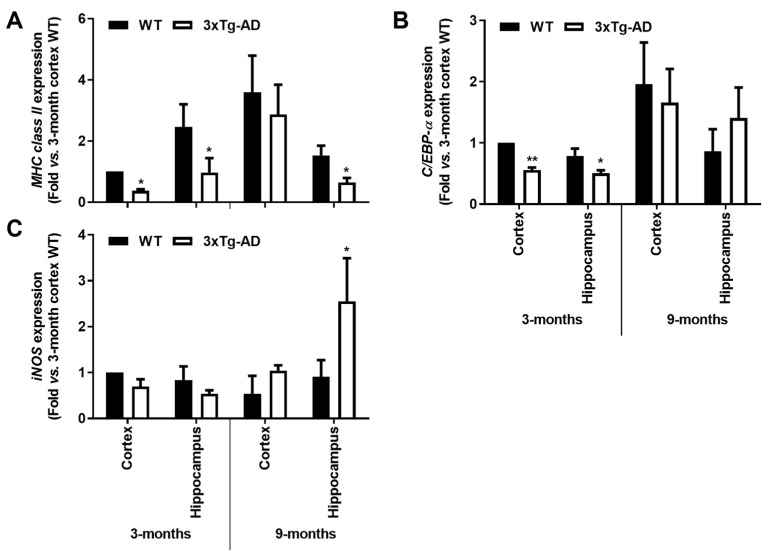
Microglia-associated *MHC-II* and *C/EBP-α* pro-inflammatory markers are downregulated in the hippocampus and cortex from the 3-month-old 3xTg-AD mice, while *iNOS* induction is apparent in 9-month-old hippocampal mouse samples. Brain samples were collected from wild-type (WT) and 3xTg-AD animals at 3 and 9 months and analyzed for (**A**) *MHC-II*, (**B**) *C/EBP-α* and (**C**) *iNOS* mRNA expression by RT-qPCR. Results are mean ± SEM, representative of *n* = 4 animals per experimental group, and are expressed as fold change vs. 3-month-old cortical tissues from the WT mice. * *p* < 0.05 and ** *p* < 0.01 vs. respective WT, two-tailed Student’s *t*-test.

At this point, we observed that both *MHC-II* and *C/EBP-α* were reduced by >55% and >35%, respectively, in both cortical and hippocampal tissues of the transgenic mice at 3-month-old (*p* < 0.05), as depicted in Figure 5A,B. Curiously, *MHC-II* was still reduced at 9 months in the 3xTg-AD hippocampus (~60%, *p* < 0.05, Figure 5A), while a slight upward trend was found for *C/EBP-α* (Figure 5B). For the expression of *iNOS*, mostly involved in the innate immune response of the conventional activated microglia [47], its predominant increase was only noticed at 9 months in the hippocampus (~2.9-fold, *p* < 0.05, Figure 5C). These data are in line with a restricted microglial phenotype early on and a more iNOS-associated cytotoxic profile later during AD progression.

**Figure 6:**
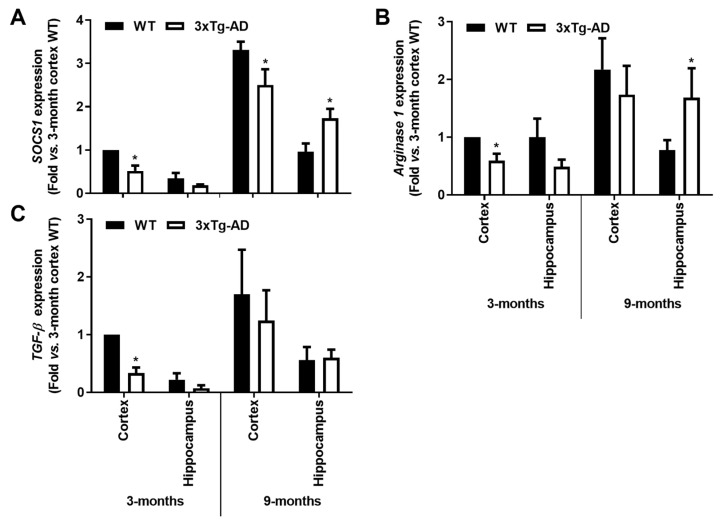
Microglia-associated *SOCS1* and *Arginase 1* anti-inflammatory markers are downregulated in the cortex from the 3-month-old 3xTg-AD mice but upregulated in the 9-month hippocampal samples, with a further *TGF-β* decrease in the first. Brain samples were collected from wild-type (WT) and 3xTg-AD animals at 3 and 9 months and analyzed for (**A**) *SOCS1*, (**B**) *Arginase 1* and (**C**) *TGF-β* mRNA expression levels by RT-qPCR. Results are expressed in graph bars as mean ± SEM, representative of *n* = 4 animals per experimental group and expressed as fold change vs. 3-month-old cortical tissues from the WT mice. * *p* < 0.05 vs. respective WT, two-tailed Student’s *t*-test.

Reinforcing the depressed microglia polarization in the transgenic mice at 3-month- old, a marked reduction of anti-inflammatory markers was also noticed, namely in cortical tissues. Indeed, reduced gene expression of *SOCS1* (<45%, *p* < 0.05), *Arginase 1* (<35%, *p* < 0.05) and *TGF-β* (<65%, *p* < 0.05) was present in cortical samples from 3xTg-AD mice at 3-month-old. In the 9-month-old animals, only *SOCS1* reduction was maintained in the cortical region. Contrasting data were obtained in the hippocampal region at this animal age, where not only the *SOCS1* was found increased (>1.7-fold, *p* < 0.05, Figure 6A), but also *Arginase 1* (>2-fold, *p* < 0.05, Figure 6B). Hippocampal increase of *Arginase 1* in the transgenic animals at 9 months may indicate a compensatory mechanism against *iNOS* induction. Overall, both microglia pro- and anti-inflammatory gene-associated markers were found reduced in the early-stage disease of 3xTg-AD mice, suggesting deactivated microglia. 

### 3.5. Inflamma-miRNAs Are Mainly Overexpressed in the Cortical Region of 3-Month-Old 3xTg-AD Mice, and Only miR-155 Remains Elevated at 9 Months

Inflamma-miRNAs, such as miR-124, miR-155 and miR-146a, are known to play a fundamental role in the regulation of microglial polarization by targeting specific molecules involved in key signaling pathways [32,48]. In conformity, it turned out to be interesting to explore the representation of such miRNAs in our samples, considering the downregulation of the unique inflammatory and microglia markers found in the early stages of AD progression in the 3xTg-AD mouse model.

**Figure 7:**
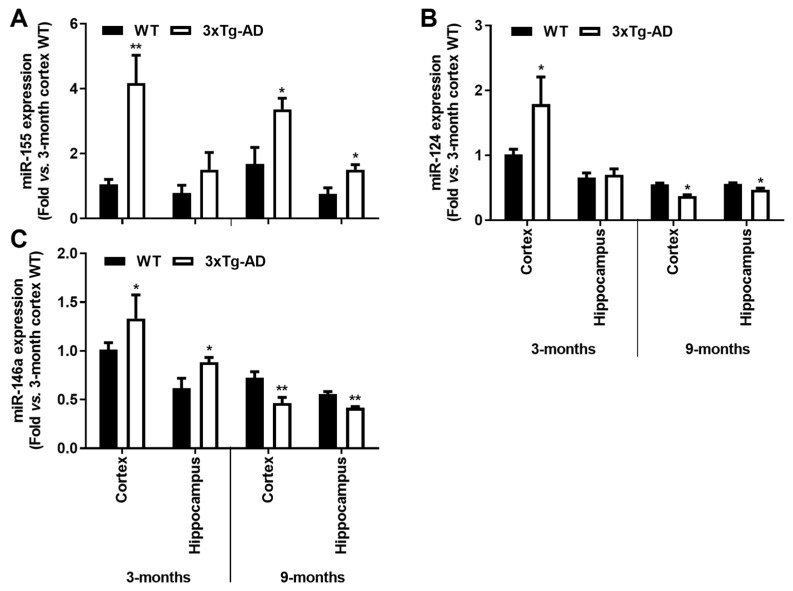
Upregulation of inflammatory-associated miRNAs is a common feature in the brain cortex at the early-AD stage of the 3xTg-AD mice, and a single elevation of miR-155 characterizes both cortex and hippocampus at 9 months. Brain samples from wild-type (WT) and 3xTg-AD animals were collected at 3 and 9 months and analyzed for (**A**) miR-155, (**B**) miR-124 and (**C**) miR-146a expression levels by RT-qPCR. Results are expressed as mean ± SEM, representative of *n* = 4 animals per experimental group and expressed as fold change vs. 3-month-old cortical tissues from the WT mice. * *p* < 0.05 and ** *p* < 0.01 vs. respective WT, two-tailed Student’s *t*-test.

Here, we observed a ~4-fold increase of miR-155 in the cortex of 3-month-old 3xTg-AD animals (*p* < 0.01) which was sustained at 9 months for both cortical and hippocampal areas (~2-fold, *p* < 0.05, Figure 7A). These results may concur with the decrease of its target *SOCS1*, above mentioned. Upregulation of miR-124 was also found in the cortical samples of 3xTg-AD mice at 3-month-old (~1.8-fold, *p* < 0.05, Figure 7B), but it was decreased at 9 months in both cortical and hippocampal regions (~30% and ~15%, respectively, *p* < 0.05). The upregulation of miR-124 in the cortex of 3xTg-AD at 3-month-old may justify the reduction we observed for its target *C/EBP-α* at this age. As for miR-146a, recently considered to oppose the pathological processes of AD [49], elevated expression was only found in the early 3 months stage in both the cortex and the hippocampus (~1.8- and 1.4-fold, respectively, *p* < 0.05, Figure 7C), significantly decreasing thereafter (~35% and ~25% for cortex and hippocampus, respectively, *p* < 0.05, Figure 7C), which may explain the increase of inflammatory molecules we noticed in 9-month-old 3xTg-AD animals. In summary, miR-155 can be sorted as an early and continuous biomarker involved in the emergence of AD pathological processes in both the cortex and the hippocampus to which the miR-146a switch from upregulated to downregulated expression levels may be accounted.

### 3.6. miR-155 Upregulation in the Cortical Region of 3xTg-AD Is Mainly Associated with a Downregulation of Genes Linked to Anti-Inflammatory and Neuroprotective Properties

**Figure 8:**
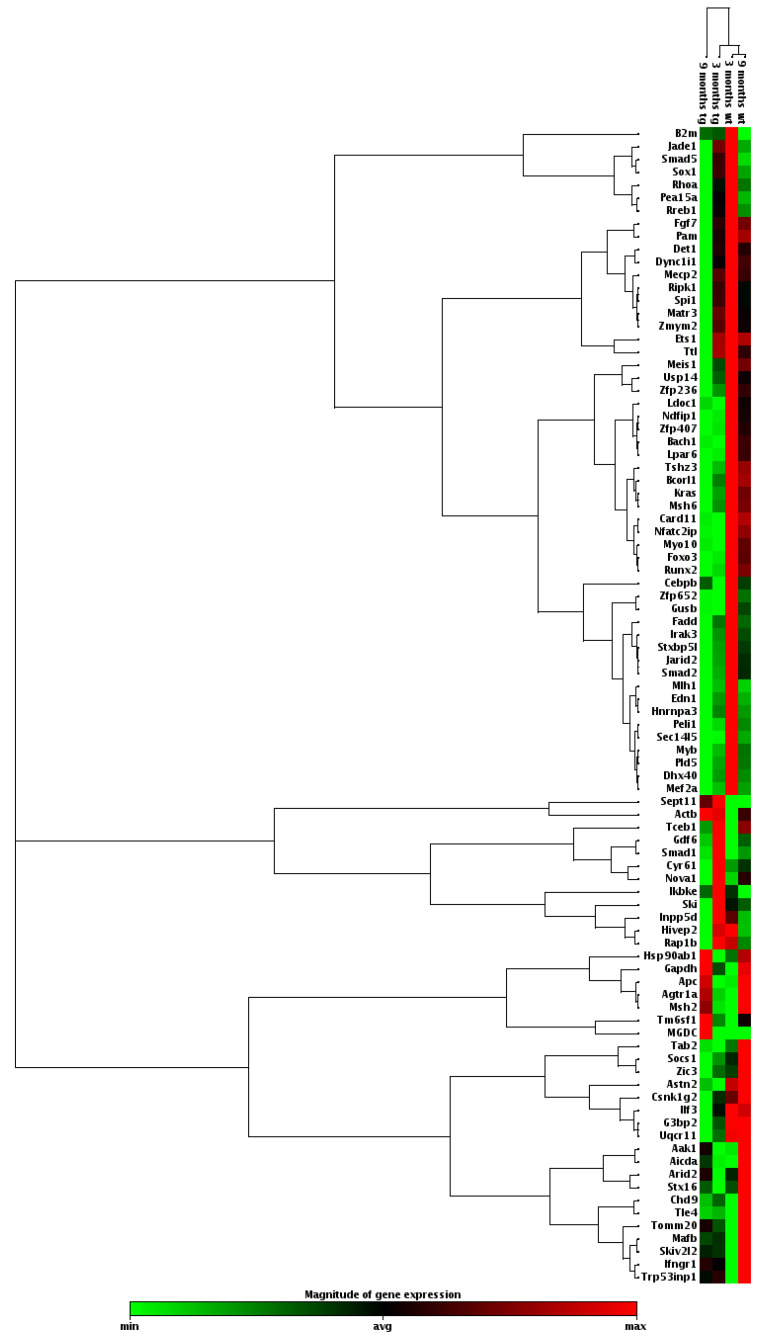
Heat map of miR-155 targets by PCR array profiling for pooled samples from 3xTg-AD mice (tg) and age-matched wild-type (WT) mice. RNA was isolated from cortical brain samples of WT and 3xTg-AD animals at 3- and 9-month-old and analyzed for miR-155 target genes by RT-qPCR. Results are expressed as magnitude of gene expression from a pool of *n* = 4 animals per experimental group.

**Figure 9:**
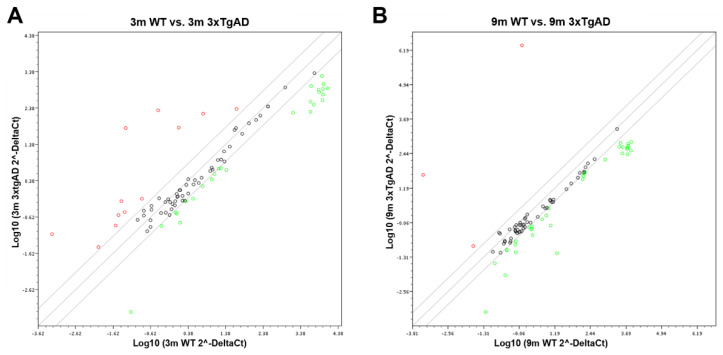
Scatter plot representation of the expression of miR-155 targets in 3xTg-AD vs. wild-type (WT) mice. RNA was isolated from cortical brain samples of WT and 3xTg-AD animals at 3- (**A**) and 9-month-old (**B**) and analyzed for the expression of miR-155 target genes by RT-qPCR. Results are expressed as Log10 gene expression from a pool of *n* = 4 animals per experimental group.

As miR-155 was found to be a potential biomarker in the early stage of the AD disease, as shown by its cortical overexpression in the 3xTg-AD mice, we decided to explore the expression profile of its known target genes. We used cortical pooled samples from 3- and 9-month-old WT and 3xTg-AD mice and performed a PCR array analysis for the miR-155 targets. Looking at the expression of some already validated and predicted targets of miR-155, we found that, aside from all the individual variation, control animals clustered together, indicating expression profile similarities, followed by 3-month-old 3xTg-AD and then the 9-month-old transgenic animals. Data suggest that changes between the 3xTg-AD mice and the age-matched WT animals increase with age. Interestingly, results also showed that the elevation of miR-155 expression levels led to both up- and downregulation of several genes (Figure 8 and Figure 9). 

The magnitude of the alterations obtained in cortical samples from the 3xTg-AD animals, as compared with age-matched WT mice, can be visualized in the heat map analysis depicted in Figure 8. Results obtained for the 9-month-old 3xTg-AD samples were those that mostly differed from the 3- and 9-month-old WT animals and included some upregulated genes but predominantly downregulated ones. However, increased, and decreased gene expression levels in the transgenic animals at 3-month-old, as compared with matched-age WT mice, were also clearly identified, and, again, a higher number of underexpressed than overexpressed genes were observed. To further explore differences between the samples collected from the cortex of 3xTg-AD mice at 3 months and 9 months with upregulated expression of miR-155, a more detailed scatter plot analysis of its targets was performed and compared with age-matched WT animals. At 3 months, the comparison of the 3xTg-AD vs. WT mice (Figure 9A) revealed the existence of 12 upregulated and 26 downregulated genes in the transgenic mice, which are listed in Table 3. *Septin11* and *Mafb* were some of those that were increased, while *Foxo3*, *Runx2* and *C/ebpβ* were found decreased.

Relative to the expression of miR-155 targets in the 9-month-old 3xTg-AD mice vs. WT animals (Figure 9B), the scatter plot analysis only identified three upregulated genes (e.g., *Septin 11*). In contrast, a large majority was found diminished. *Runx2*, *Foxo3* and *Socs1* were some examples among the 35 genes listed in Table 4 that we found to be downregulated. The *Socs1* decrease corroborates our previous finding shown in Figure 6 for the 9-month-old cortical samples. Most of the downregulated genes are associated with deregulated neuro-immune interactions, microglia malfunction, synaptic connectivity deficits and amyloid pathology.

## 4. Discussion

Experiments in this study were established to identify initial biomarkers of neuro-immune deregulation and microglia malfunction using the cortex and the hippocampus of the 3xTg-AD mouse model of AD, which are two of the brain areas most affected by AD pathology [50]. Here, we observed that inflammatory mediators and microglia homeostatic/reactive markers are depressed early and can be detected at 3 months in our AD mouse model, suggesting that microglia and probably also astrocytes refrain their reactivity to hold the disease onset. In preceding studies, we found that microglia and astrocyte pro-inflammatory and reactive markers were also decreased at the presymptomatic stage in the *SOD1G3A* mice, a model commonly used to discriminate the pathological events of amyotrophic lateral sclerosis [45]. Actually, hypometabolism and bioenergetic deficits have been described as early emerging events in AD prodromal stage [51] and impaired glucose metabolism was found in the young 3xTg-AD mice [52]. This corroborates our present data showing a deficiency in *CX3CR1*, also obtained in AD mouse models in the past [53], and in *MFG-E8*, documented to be important in reversing microglia-induced astrocyte neurotoxicity [54]. Prior studies in our 3xTg-AD mouse model did not identify differences in the number or phenotype of microglia and astrocytes at 3-month-old, based on IBA-1 and GFAP immunostaining [16]. Validating the restricted neuro-immune balance in the 3-month-old 3xTg-AD mice it is also the low expression of HMGB1 protein levels, an alarmin indicated as mediating neuroinflammatory responses in brain injuries [55]. A decrease of HMGB1 was particularly noticed in the cortical region of the 3-month-old 3xTg-AD mice, where we also found an overexpression of IL-4 that was proposed as an endogenous defense mechanism in other studies [56]. Together with that, the IL-10 deficiency we also identified in the brain cortex at the early stage may additionally sustain the constrained neuro-immune balance once its decreased levels are associated with the preservation of synaptic integrity and cognitive function in the APP/PS1+ Il10^−^/^−^ mouse model [57].

Once past reports demonstrated the existence of at least a sub-population of microglial cells with a restrictive phenotype [28], the gene assembly identified to be reduced at the early disease stage, together with miR-146a downregulation, validates the 3-month-old 3xTg-AD mouse as a good model to further explore the consequences of such homeostatic imbalance if immune-challenged. The cross-temporal upregulation of miR-155 can be sorted in this study as a most promising AD biomarker in the prodromal stage, requesting future studies to address this possibility in patients. miR-155 overexpression was also shown to trigger an increased number of downregulated genes, at least in the cortical region of the transgenic mice. It should be noted that such a neuro-immune depressed network may be considered a negative factor if reparative or insult-related responses are required. In such a case, the targeting of inhibitory checkpoint activation can have benefits against progressive neuropathological alterations in AD.

AD is a continuing and irreversible neurodegenerative disorder that gradually reduces memory and thinking skills, leading to the inability to perform simple daily tasks [23]. Inflammation in AD has been associated with circulating pro-inflammatory cytokines by stress and cellular senescence [58] and risk genes [59], mainly those expressed in microglia [60]. Microglial-derived cytokines are described to be neurotoxic, to support a chronic inflammatory milieu and to contribute to disease progression [26]. Reports also indicate that the secreted molecules can lead to divergent effects depending on the extent and type of microglial activation, thus proposing that a fine equilibrium should be preserved [61]. Thus, it is extremely important to understand the pathogenic processes in AD and to discover targets that may help in new drug discovery toward microglia homeostasis.

Animal models are important tools to identify the drivers behind the onset and progression of AD-related cognitive decline, as well as to assay potential pharmacological interventions. So, in our work, we used the 3xTg-AD mouse model of AD, which progressively develops both Aβ plaques and neurofibrillary tangles with a temporal and spatial distribution that closely mimics the human AD brain [14,15,38]. The sequence of neuropathological development in this model suggests that Aβ is the initiating trigger of cognitive decline [14]. The 3xTg-AD mice model develops AD pathology in an age-dependent and progressive manner, ahead of the associated behavioral changes [62], similar to human patients [63]. Intraneuronal Aβ accumulation was reported in the cortex of these animals at around 3-month-old, spreading to the hippocampus and amygdala by 6 months, together with diffuse amyloid plaques in the cerebral cortex [15,20]. However, more recently, Aβ accumulation was only reported in the hippocampus for 6-month-old animals and in the cortex for 12-month-old 3xTg-AD mice [38]. However, widespread senile plaques were only detected at 12 months [14,64], coupled with the emergence of conformational changes in tau [15]. Detection of APP in the 3xTg-AD animal at 3-, 6- and 9-month-old, although at levels below those reported [15], is in accordance with previous data in the same animal colony [16] and may correspond to the β-secretase-derived C-terminal fragment of APP found in the 3xTg-AD hippocampi [65]. In other studies, changes in APP levels were not found at 5–7 months, and not even observed between 12 and 18 months in the same model [66], representing either variation in the APP expression levels or in the antibody used to detect it among studies. Moreover, APP enrichment in exosomes was outlined as a sorting mechanism into the brain extracellular space [67].

An increased density of GFAP immunoreactive astrocytes and IBA1 immunoreactive microglia, as well as upregulation of inflammatory markers, were shown in 3xTg-AD mice compared with WT at 3 [68] and at 6/7 months [21,22,38], suggesting that it may be a response to Aβ accumulation. Curiously, we found a marked downregulation of the microglia homeostatic/reactive markers, *CD11b, CX3CR1* and *MFG-E8*, at 3 months in the cortex and hippocampus of the 3xTg-AD mice, as compared with age-matched WT animals. Our findings corroborate the previous report by Janelsins and colleagues, showing decreased F4/80-positive microglia in the hippocampus of 3- and 6-month-old 3xTg-AD mice [69] and that of Rodriguez and colleagues mentioning an increased density of both resting/ramified and activated microglia in the hippocampus of 9-month-old 3xTg-AD animals prior to senile plaque spread [64]. This increased density of ramified microglia may be a preparation stage to refrain the Aβ-associated damage. Furthermore, a recent study showed that microglia reactivity is enhanced in the 3xTg-AD mice only following *T. gondii* infection at 5-, 6- or 11/12-month-old [70]. Finally, it should be noted that microglia have regional and age-dependent variations in microglial phenotypes and that the definition of a subtype requires the analysis of a set of genes [71]. Density of both resting and activated microglia was previously reported to significantly increase in 12- and 17-month-old 3xTg-AD mouse hippocampi but not at 9-month-old, only based on polyclonal affinity-purified rat antiserum raised against CD11b (MAC-1)-IR density with no further validation by gene signature [64]. Once increased, Cd11b microglia were observed near Aβ plaques by immunostaining [64,72]; such finding may justify the unchanged gene expression levels we obtained in our total homogenates. Moreover, it is documented that common microglial markers, such IBA1 and CD11b, stain both resting and activated cells and their determination was shown to be heterogeneous and to not demonstrate a consistent elevation in samples from the AD brain [73]. Moreover, CD11b was not found upregulated in the microglia of double APP/PS1 transgenic mice, while it was upregulated in the PS19 mouse model in the 3-month-old animals [72], corroborating the wide range of CD11b expression levels among AD models and its environment-dependent changes.

It has been described that microglia activation promotes the release of potentially cytotoxic molecules, which contribute to the onset and/or progression of AD [26]. Several cytokines, including IL-1β, IL-18 and TNF-α, were shown to be overexpressed in the AD brain [23,74]. In a previous study, we found that such pro-inflammatory cytokines are secreted by microglia upon the interaction with Aβ1-42 oligomers and fibrils [30]. However, expression levels in AD mouse models were usually found to be similar to WT animals [38,40], or even to be reduced [16,46]. In the present study, the diminished expression of *CD11b*, *CX3CR1*, *MFG-E8*, *TNF-α* and *IL-1β* gene levels in the brain cortices and hippocampi, with a more robust decrease for the last two genes, together with the decrease of HMGB1 protein and the pro-inflammatory genes *IL-6* and *IL-18* only in the cortex, validate the presence of an immunosuppressed microglia in the young 3xTg-AD animals and their failure in preventing AD plaque pathology [75]. The reduction of hippocampal expression levels of *IL-6, TNF-α* and *IL-1β* was also observed in the same 3xTg-AD at 3-month-old [16,69]. Moreover, while *IL-10* was reduced in the cortex of 3-month-old 3xTg-AD animals, *IL-4* was found enhanced. Interestingly, administration of IL-4 into an amyotrophic lateral sclerosis animal model was reported to induce microglia to adopt a slowly proliferating phenotype [76], which may corroborate a skewed microglia reactivity in these young animals. Curiously, *IL-4* expression was deficient in the cortex of 9-month-old 3xTg-AD animals in opposition to upregulation of inflammatory markers, such as *IL-1β* and *HMGB1*. This inverse correlation was previously reported in the 3xTg-AD mouse model where hyperbaric oxygen therapy was shown to reduce gliosis in parallel with the decrease of pro-inflammatory cytokines (IL-1β and TNF-α) and the increase of Arginase 1 and anti-inflammatory cytokines (IL-4 and IL-10) [77]. 

In contrast, enhanced production of TNF-α and IL-1β was detected in the *APP/PS1ΔE9* mouse model at 10-month-old [40] and upregulation of inflammatory genes at 4-6 months in the 5xFAD animals, namely in the hippocampus [39], which may, eventually, be linked to Aβ accumulation. Indeed, we found upregulated gene expression levels of *TNF-α*, *IL-1β, IL-6* and *HMGB1* in the hippocampus, with the last also increased in the cortex, in our 3xTg-AD mouse model. TNF-α and IL-1β have been described for a long time as mediators of AD pathogenesis [78]. HMGB1 was reported to accumulate extracellularly on Aβ plaques, being the protein level increased in AD brains [79]. More recently, HMGB1 was implicated in the pathogenesis of AD by causing a microglial Aβ phagocytosis dysfunction [80] and neurite degeneration [81]. Distinct microglia phenotypes were found in the frontal cortex samples of AD patients and associated with the presence of different Aβ species [82]. One with high expression levels of *IL-1β*, *TNF-α* and *IL-12A* typical of a pro-inflammatory phenotype related with oligomeric Aβ, the other located near senile plaques with upregulation of *IL-1ra*, *Arginase 1* and *FIZZ*, is characteristic of a reparative phenotype. 

MHC-II positive microglia participate in the adaptive immune response, though it is questionable whether it is a marker of amoeboid/activated or ramified/homeostatic microglia [83]. Unchanged levels of *MHC-II* gene expression were found in the cortex of the AD mouse model at 9 months. Decreased expression was present in the hippocampus at both 3 and 9 months and in the cortex only at 3 months. Other studies report no changes in the *APP/PS1ΔE9* model [40]. *C/EBP-α* was described to regulate the transcription of the *MHC-II* gene in murine microglia [84]. Interestingly, it was found decreased in the cortex and hippocampus of 3-month-old 3xTg-AD animals but unaffected at 9 months. *iNOS*, the typical pro-inflammatory marker, was only upregulated at the hippocampus in the older animals, probably exerting cytotoxic effects since it uses arginine to produce nitric oxide [47]. Elevation of microglial iNOS was also observed in the *APP/PS1* and *APP/PS1ΔE9* models at 10-15 months [40]. 

It has been reported that SOCS1 is involved in the shift of macrophages from a pro-inflammatory to a reparative state [85], and SOCS1 was shown to attenuate Aβ-induced inflammation in a microglial murine cell line [86]. Decreased levels of *SOCS1* were noticed in the cortex at both 3xTg-AD animal ages, though it was increased in the hippocampus at 9 months. In addition, *Arginase 1* was shown to be upregulated in 9-months 3xTg-AD hippocampi and revealed to reduce Aβ plaque formation in conditions of IL-1β-dependent inflammation [87], as happened in our model with upregulated hippocampal *IL-1β* at this stage. *TGF-β* downregulation observed in the cortex of the young transgenic mice may facilitate Aβ-induced microglial chemotaxis, adding distinct microglial responses in the 3xTg-AD mice [88]. 

miRNAs regulate intracellular processes by targeting multiple mRNA molecules simultaneously, thus controlling immune cell phenotypes [16]. Deregulation of innate immune- and neuroinflammation-related miRNAs were identified in patient AD brains [89,90], together with disturbed microglia responses [91]. Here, we show that miR-146a is overexpressed as early as 3 months in the cortex and hippocampus of the 3xTg-AD mice, while similar findings for miR-155 and miR-124 only occurred in the cortical brain. Elevation of these miRNAs in AD patients and AD mouse models was previously documented [16,34,90,92,93,94,95]. Such early upregulation may represent compensatory mechanisms over the depressed neuro-immune axis in the transgenic mice at 3-month-old. Elevation of miR-155 can derive from upregulated *HMGB1* [96] and may lead to decreased levels of *SOCS1*, its well-known target [97,98]. Indeed, it was recently shown that chronic treatment of 3xTg-AD mice with an anti-TNFSF10 monoclonal antibody inhibits retinal expression of miR-155, leading to SOCS1 upregulation [99]. A visible increase of miR-155 labelling was previously observed in the hippocampus region in 3-month-old 3xTg-AD mice, with a further strong enhancement at 12 months [16]. A decrease of miR-124 at 9 months may result from some neurodegenerative processes [32] and facilitate the pro-inflammatory status we found in the 3xTg-AD mice at such an age. Actually, Ponomarev and colleagues reported that the upregulation of miR-124 promoted microglia reparative processes by causing the downregulation of inflammatory-associated markers, such as TNF-α, iNOS and MHC-II [84]. Reduction of miR-146a contrasts with that of miR-155 upregulation and reinforces their opposite roles in immune response [100], pushing toward the pro-inflammatory signature and the increased expression of associated genes observed in the 3xTg-AD mice at 9-month-old. 

The elevation of the miR-155 expression in the cortex at both 3 and 9 months was never explored in the 3xTg-AD mice for the alterations that it may induce in its multiple target genes. Looking at the expression of some miR-155-validated and predicted targets, we found that, aside from all the individual variation, control animals clustered together indicating expression profile similarities, followed by 3-month-old 3xTg-AD and then the 9-month-old transgenic animal. Data suggest that changes between the 3xTg-AD mice and the age-matched WT animals increase with age. As expected, most of the target genes were found decreased, i.e., 26 genes at 3 months and 35 genes at 9 months. However, some upregulation was also noticed, mainly in the younger transgenic animals, probably related with indirect effects to distal mRNAs. *Septin11* was elevated at both ages and the *Mafb* gene only at 3 months in the 3xTg-AD mice. Genes from the Septin family are involved in vesicle trafficking and synaptic connectivity [101] and favor the β-amyloidogenic processing of APP through BACE1 accumulation [102], while the *MafB* gene is implicated in microglia inflammatory control [103]. MafB is a transcription factor involved in microglia differentiation [104] with an important role in the maintenance of homeostasis in adulthood if one considers that the knockout of microglia *Mafb* induces the expression of inflammation-related pathways [103]. So, *MafB* upregulation may be linked to the decrease of the inflammatory markers obtained in the 3-month-old transgenic mice. Relative to the downregulated genes, the *C/EBP-β* gene is, indeed, a direct target of miR-155 in this AD model [16], and its reduction is linked to a decrease in cell apoptosis [105] and delayed cell senescence [106]. On the other hand, the downregulation of *Run2*, at both 3 and 9 months in the transgenic animals may lead to a reduction in microglia phagocytic ability [107]. Relative to the *Foxo3* gene, considered to be associated to longevity [108], its downregulation in 5xFAD mice also showed to exacerbate Aβ pathology and synapse loss by hampering the uptake of Aβ by astrocytes and microglia [109]. These findings open new avenues for further target evaluation concerning common and causal distinct stages of microglia and astrocyte dysfunctionalities in AD onset and progression. By showing divergent microglia phenotypes and inflammatory-associated features across AD state trajectory, the 3xTg-AD mice may be the appropriate model for fine-tuning the genetic signature of the brain’s immune cells at the different unbalanced homeostatic windows that characterize AD progression.

## 5. Conclusions

Overall, our results highlight a restricted microglia phenotype at both the cortex and hippocampus in the 3-month-old 3xTg-AD mice. This loss in microglia reactivity may be responsible for the reduced levels of tissue and microglia inflammatory mediators at such an age. Such homeostatic deregulation can be interpreted as a defensive mechanism to prevent Aβ accumulation, in accordance with the global downregulation of the expression of genes encoding proteins that are metastable to aggregation, as observed in another study [110]. However, the upregulation of miRNAs, mainly of miR-155, may counterbalance the defective neuro-immune response, though the downregulated miR-155 target genes seem to have dual intervention. On the one hand, they favor Aβ pathology by increased APP processing and decreased Aβ uptake. On the other hand, they support cell survival. Reduced expression levels of miR-146a and miR-124, considered to be anti-inflammatory [32], are believed to contribute to the upregulated immune system crosstalk we observed in 9-month-old 3xTg-AD mice. Upregulated inflammatory mediators predominate at the hippocampus at such an age. These age-dependent neuroinflammatory changes may be critical to the cognitive decline [111] and explain the controversies about the therapeutic efficacy of non-steroidal anti-inflammatory drugs (NSAIDs) in AD [112,113]. In summary, if the homeostatic microglia phenotype is lost early, the miR-155 overexpression and the upregulated APP processing mechanisms are among the first pathological features preceding AD onset. Future studies should explore which modulators for the identified targets are able to recover homeostasis and prevent the course of the disease.

## Figures and Tables

**Table 1:** List of pairs of primers used to determine gene expression by RT-qPCR assays.

Gene	Primer	Sequence
Arginase1	Sense	5′-CTTGGCTTGCTTCGGAACTC-3′
	Anti-sense	5′-GGAGAAGGCGTTTGCTTAGTTC-3′
β-actin	Sense	5′-GCTCCGGCATGTGCAA-3′
	Anti-sense	5′-AGGATCTTCATGAGGTAGT-3′
CD11b	Sense	5′-CAGATCAACAATGTGACCGTATGGG-3′
	Anti-sense	5′-CATCATGTCCTTGTACTGCCGCTTG-3′
C/EBP-α	Sense	5′-AGCTTACAACAGGCCAGGTTTC-3′
	Anti-sense	5′-CGGCTGGCGACATACAGTAC-3′
CX3CR1	Sense	5′-GTGGTGCTGACAAAGCTTGGA-3′
	Anti-sense	5′-TCACTGGGTGCCATCGTAAGAA-3′
HMGB1	Sense	5′-CTCAGAGAGGTGGAAGACCATGT-3′
	Anti-sense	5′-GGGATGTAGGTTTTCATTTCTCTTTC-3′
IL-1β	Sense	5′-CAGGCTCCGAGATGAACAAC-3′
	Anti-sense	5′-GGTGGAGAGCTTTCAGCTCATA-3′
IL-4	Sense	5′-TCGGCATTTTGAACGAGGTC-3′
	Anti-sense	5′-GAAAAGCCCGAAAGAGTCTC-3′
IL-10	Sense	5′-ATGCTGCCTGCTCTTACTGA-3′
	Anti-sense	5′-GCAGCTCTAGGAGCATGTGG-3′
IL-18	Sense	5′-TGGTTCCATGCTTTCTGGACTCCT-3′
	Anti-sense	5′-TTCCTGGGCCAAGAGGAAGTG-3′
iNOS	Sense	5′-ACCCACATCTGGCAGAATGAG-3′
	Anti-sense	5′-AGCCATGACCTTTCGCATTAG-3′
MFG-E8	Sense	5′-AGCCTGAATGGTAGGGTTGG-3′
	Anti-sense	5′-GAGACTGCATCCTGCAACCA-3′
MHC-II	Sense	5′-TGGGCACCATCTTCATCATTC-3′
	Anti-sense	5′-GGTCACCCAGCACACCACTT-3′
SOCS1	Sense	5′-CACCTTCTTGGTGCGCG-3′
	Anti-sense	5′-AAGCCATCTTCACGCTGAGC-3′
TGF-β	Sense	5′-CAGAGCTGCGCTTGCAGAG-3′
	Anti-sense	5′-GTCAGCAGCCGGTTACCAAG-3′
TNF-α	Sense	5′-TACTGAACTTCGGGGTGATTGGTCC-3′
	Anti-sense	5′-CAGCCTTGTCCCTTGAAGAGAACC-3′

All primers were purchased from Thermo Fisher Scientific, Waltham, MA, USA. CD11, cluster of differentiation-11; C/EBP-α, CCAAT enhancer binding protein-α; CX3CR1, C-X3-C Motif Chemokine Receptor 1; HMGB1, high mobility group box 1; IL-1, interleukin-1; iNOS, inducible nitric oxide synthase; MFG-E8, milk fat globule-EGF factor 8; MHC-II, major histocompatibility complex class II; SOCS, suppressor of cytokine signaling; TGF-β, transforming growth factor-β; TNF-α, tumor necrosis factor-α.

**Table 2:** List of target sequences used to determine miRNA expression in RT-qPCR.

miRs	Target Sequence (5′-3′)
miR-124-3p	UAAGGCACGCGGUGAAUGCC
miR-146a-5p	UGAGAACUGAAUUCCAUGGGUU
miR-155-5p	UUAAUGCUAAUUGUGAUAGGGGU

All primers were purchased from Qiagen, Hilden, Germany. SNORD110 and RNU1A1 were used as a reference gene (human, mouse and rat); UniSp6—RNA spike-in control was used to monitor PCR efficiency. miR, miRNA.

**Table 3:** Expression of miR-155 target genes in cortical samples from 3-month-old 3xTg-AD mice vs. respective wild-type (WT) animals.

Genes Upregulated		Genes Downregulated			
Gene	Fold Regulation	Gene	Fold Regulation	Gene	Fold Regulation
***Septin 11***	**1314.551**	*Aak1*	−122.87	*Kras*	−5.327
*Tceb1*	538.838	*Zfp652*	−23.544	*Stxbp5l*	−4.763
*Tm6sf1*	143.186	*Sec14l5*	−23.476	***C/ebpb***	**−4.676**
***Mafb***	**51.209**	*Ldoc1*	−17.99	*Mlh1*	−4.22
*Gdf6*	28.071	*Nfatc2ip*	−17.159	*Peli1*	−3.995
*Ifngr1*	16.511	*Ndfip1*	−15.305	*Bach1*	−3.84
*Gapdh*	7.956	*Card11*	−12.27	*Pld5*	−3.5
*Skiv2l2*	6.776	*Lpar6*	−11.942	*Hnmpa3*	−3.46
*Smad1*	5.384	***Foxo3***	**−11.472**	*Irak3*	−3.412
*Cyr61*	5.016	***Runx2***	**−9.585**	*Myo10*	−3.401
*Nova1*	4.829	*Zfp407*	−9.067	*Zfp236*	−3.261
*Aicda*	3.679	*Gusb*	−8.814	*Mef2a*	−3.171
		*Tab2*	−8.525	*Fadd*	−3.073

**Table 4:** Expression of miR-155 target genes in cortical samples from 9-month-old 3xTg-AD mice vs. respective wild-type (WT) animals.

Genes Upregulated		Genes Downregulated			
Gene	Fold Regulation	Gene	Fold Regulation	Gene	Fold Regulation
*MGDC*	2,160,132.239	*Kras*	−268.814	*Zfp236*	−5.144
***Septin11***	**131,567.175**	*Cyr61*	−113.564	*Uqcr11*	−4.848
*Ikbke*	5.699	*Mecp2*	−26.774	*Fadd*	−4.839
		***Runx2***	**−21.285**	*Dync1i1*	−4.614
		*Ndfip1*	−20.299	*Ldoc1*	−4.576
		*Fgf7*	−16.183	*Nova1*	−4.503
		***Foxo3***	**−13.309**	***Socs1***	**−4.497**
		*Stxbp5l*	−12.971	*Hnmpa3*	−3.986
		*G3bp2*	−12.318	*Tceb1*	−3.966
		*Lpar6*	−11.39	*Irak3*	−3.953
		*Tab2*	−11.381	*Ski*	−3.715
		*Nfatc2ip*	−10.592	*Gusb*	−3.605
		*Zfp407*	−9.474	*Pea15a*	−3.539
		*Det1*	−9.026	*Ets1*	−3.248
		*Rreb1*	−8.283	*Bcorl1*	−3.247
		*Card11*	−7.964	*Zic3*	−3.118
		*Zfp652*	−5.937	*Tle4*	−3.088
		*Sec14l5*	−5.397		

## Data Availability

The data presented in this study are available on request from the corresponding authors.
